# Dissolution and uniformity of content of tablets developed with extract of *Ximenia americana L*.

**DOI:** 10.1371/journal.pone.0197323

**Published:** 2018-05-24

**Authors:** Cleildo P. Santana, Francinalva D. Medeiros, Lidiane P. Correia, Paulo Henrique G. D. Diniz, Germano Véras, Ana Cláudia D. Medeiros

**Affiliations:** 1 Laboratório de Desenvolvimento e Ensaios de Medicamentos, Centro de Ciências Biológicas e da Saúde, Universidade Estadual da Paraíba, Campina Grande, Paraíba, Brasil; 2 Departamento de Farmácia, Centro de Ciências Biológicas e da Saúde, Universidade Estadual da Paraíba, Campina Grande, Paraíba, Brasil; 3 Laboratório de Química Analítica e Quimiometria, Universidade Estadual da Paraíba, Campina Grande, Paraíba, Brasil; Institute of Materials Science, GERMANY

## Abstract

Herbal medicines currently represent an important part of the world pharmaceutical market, which shows growing interest in the use of herbal medicines. However, the production of such medicines involves a complex series of steps, which determine the production viability and the quality of the final product. *Ximenia americana* L. is a plant occurring in several regions of the world, with well-known and applied medicinal properties. Thus, the aim of this work was to develop and evaluate the physical and physical-chemical quality of tablets produced with *X*. *americana* L. extract. The extract was spray-dried from a hydroethanolic extractive solution and characterized as to its phytochemical composition. The chemical marker was determined and quantified using validated chromatographic methods. These methods indicated the presence of gallic acid at a concentration of 1.61 mg g^-1^. Formulations were proposed and analyzed for their flow and compaction properties. The best formulation was used to obtain a batch of tablets, which was evaluated for its quality characteristics and showed to be within the pharmacopoeial specifications for average weight, hardness, friability, and disintegration time. The dissolution profile of the tablets produced was obtained, showing the release of about 70% of the vegetable extract content within 30 minutes. Results showed that it was possible to obtain herbal tablets containing a high content of vegetal extract by direct compression, developing a rapid process of formulation and production and guaranteeing the quality characteristics of the final product.

## Introduction

*Ximenia americana* L. is a plant found in South America, Central America, Africa, India, and New Zealand, and its medicinal properties have long been known and used by local populations. In the Brazilian semiarid region, traditional medicine designates this plant for the treatment of internal organ inflammation, toothaches, menstrual cramps, and as an antiseptic and an antimicrobial [1,2,3].

Dried plant extracts are used by the pharmaceutical industry as plant active pharmaceutical ingredients (PAFI) for herbal medicines in various dosage forms. However, in order to guarantee the quality characteristics of these extracts, they must be standardized. A method used for standardization is the qualitative and quantitative determination of one or more compounds of the PAFI, which are called chemical markers. Information on the presence and quantity of these compounds in the PAFI allows the establishment of important parameters of quality control for herbal medicines [4,5].

Tablets are solid pharmaceutical forms, obtained through the aggregation of powders by applying pressure. The direct compression method is considered the most efficient for the production of herbal tablets, because it involves fewer processing steps and therefore less cost and time. However, this type of active pharmaceutical ingredient usually has unsatisfactory flow, compressibility, and hygroscopicity characteristics [6]. An adequate study of pharmaceutical adjuvants is necessary in order to ensure suitable technologic properties for formulation compression.

Evaluation of the tablets’ quality is essential in ensuring the use of the medicinal product under conditions that guarantee the safety, therapeutic efficacy, and quality of the product throughout the valid time; therefore, physical and chemical methods must be taken into account [7].

Thus, the aim of this work was to develop and evaluate the physical and physical-chemical quality of tablets produced with *X*. *americana* L. extract.

## Material and methods

### Ethics statement

The plant was collected and no specific permits were required. This access was registered in the National System of Genetic Heritage and Associated Traditional Knowledge (SISGEN) under the number AFD885A. The location where the plant was collected was not privately-owned or protected in any way and did not involve endangered or protected species.

All data were obtained by the authors, and are accessible for all in the same manner as the authors, without special access privileges. The data don’t contain potentially identifying or sensitive information.

### Obtaining the *X*. *americana* extract

*Ximenia americana* L. bark was collected in the semi-arid region of the Paraíba state (7°08'33.8" S; 36°06'2 1.1" W) in Brazil. The voucher specimen was prepared and identified at the Professor Jayme Coelho de Morais herbarium (PRU), Federal University of Paraiba, under the number EAN-100493. The collected plant material was dried in a forced ventilation oven at 40 °C and pulverized in a knife mill with particle size of about 10 mesh. Two hundred grams of the plant drug were then subjected to extraction by cold maceration in 1000 mL of a hydroethanolic solution (70% v/v) for five days. The extractive solution was nebulized in a spray dryer, with inlet temperature of 120 °C and outlet temperature between 90 and 95 °C, under solution flow of 3.0 mL min^-1^. In the drying process, Aerosil 200^®^ was used as a drying adjuvant at the proportion of 20% (m/m) of the extract dry residue.

### Fingerprint of the *X*. *americana*

The extract was evaluated for its phytochemical composition. Polyphenols and total flavonoids were quantified by spectrophotometry in the UV-visible region, following the methods described by Chaves et al. [8]; condensed tannins were quantified using the method described by Makkar and Becker [9]. The tests were performed using a Shimadzu UVmini-1240 spectrophotometer.

The chemical marker of the extract was determined using a high performance liquid chromatograph (HPLC) (Dionex) coupled to a diode array detector (DAD), with scanning in the UV-visible region. The chemical marker was quantified using a high performance liquid chromatograph (HPLC) (Shimadzu) coupled to a simple UV-visible detector, monitoring the wavelength of 270 nm.

For both determination and quantification, a Gemini NX C18 (Phenomenex) (250 mm x 4.6 mm, **5 μm**) chromatographic column was used at 30 °C. The mobile phase consisted of a mixture of acetic acid at 0.1% (v/v) (A) and methanol (B) in an elution system, according to the following sequence: 0–3 minutes—10% B isocratic; 3–10 minutes—gradient up to 12% B; 10–12 min—gradient up to 90% B; 12–17 minutes—90% B isocratic; 17–20 minutes—10% B isocratic. The samples were pre-dissolved in a methanol solution (50% v/v) for analysis.

The chromatographic method was validated by analysis of the following parameters: specificity, linearity, repeatability, intermediate precision, accuracy, and detection and quantification limits [10].

#### Specificity

Specificity of the separation method was evaluated in the HPLC-DAD analyses. The analysis was performed by comparing the retention times of the extract chromatographic peaks with those of chromatographic standards (Sigma-Aldrich), and by determining the chromatographic peak purity via comparing the UV-Vis spectra at 50 and 100% of peak height. Specificity assessment also took into account the possible interference of pharmaceutical excipients in the separation method.

#### Linearity

The method linearity was analyzed by standard addition, constructing calibration curves of the extract at a fixed concentration (5.00 mg mL^-1^) and adding increasing concentrations of the chromatographic standard. The calibration curves were analyzed by evaluation of four parameters—determination coefficient (R^2^), residue analysis, lack of fit test, and regression significance test—using F values for 5% significance.

#### Repeatability and intermediate precision

Repeatability was evaluated by analyzing extract solutions (5.00 mg mL^-1^), with increasing concentrations of standard, at three concentration levels: low, medium, and high (2.5, 15.0, and 25.0 μg mL^-1^, respectively), in the presence and the absence of the formulation excipient matrix. For the intermediate precision evaluation, these samples were analyzed on three different days. All samples were analyzed in triplicate.

#### Accuracy

Accuracy was evaluated by comparing the chemical marker concentrations determined in extract solutions at three concentration levels (2.5, 15.0, and 25.0 μg mL^-1^) with their theoric concentration values.

#### Detection and quantification limits

The detection and quantification limits were determined from the standard deviation of the chromatogram baseline noise.

### Formulation development

#### Preformulation studies

The tablet was developed after the compatibility study between the extract of *X*. *americana* (AMCA) and pharmaceutical excipients, as described by Santana et al. [11]. Binary mixtures were prepared from physical mixtures of the extract with the following pharmaceutical excipients: corn starch, lactose, microcrystalline cellulose PH 101 and 102, magnesium stearate, polyvinyl pyrrolidone K-30 (PVP K-30), talc, colloidal silicon dioxide, sodium starch glycolate, pregelatinized starch, and sodium croscarmellose.

#### Repose angle determination

The static repose angle was determined based on the fixed height of a glass funnel. Ten grams of the tablet formulation powder were passed through the funnel so as to form a cone on a millimeter paper. The tangent of the angle of repose was calculated by the ratio *tgα = H/R*, where α is the angle of repose, H is the height, and R is the radius of the cone. The results were calculated by the average of five determinations. The flow time was measured in seconds and was also determined by the average of five measurements.

#### Determination of bulk and compaction densities, Hausner ratio, Carr index, and densification index

For the evaluation of the titular parameters, 10 g samples of the formulation (m) were used. The samples were poured into a graduated cylinder, which determined the bulk volume (BV). The cylinder was then subjected to 10, 500, and 1250 sequential falls, determining the volumes V_10_, V_500_, and V_1250_, respectively. The test was continued in sequences of 1250 falls until the difference between two subsequent readings was less than or equal to 0.1 mL, which was considered the compression volume (CV). The test was performed fivefold. The data obtained made possible the calculations of the bulk density (BD), compaction density (CD), Hausner ratio (HR), Carr index (CI), and densification index (DI):

$$BD=\frac{m}{BV}$$ (1)

$$CD=\frac{m}{CV}$$ (2)

$$HR=\frac{CD}{BD}$$ (3)

$$CI=\frac{CD-BD}{CD}x100$$ (4)

$$DI=V_{10}-V_{500}$$ (5)

#### Development and standardization of *X*. *americana* tablets

The compatibility study carried out previously, and the tests described above, allowed for the choice of a formulation that was directly compressed using a compressor (Lemaq Monopress LM-1). A batch of 100 tablets of about 300 mg was obtained and evaluated for its quality parameters by means of testing average weight, hardness, friability, and disintegration time, according to the methodology described in The United States Pharmacopoeia [12].

To evaluate the extract content uniformity, five tablets were dissolved in a 50% (v/v) methanol solution; the corresponding chemical marker content for the extract was determined by HPLC, according to the methodology described in section 2.2. The results were compared with the expected extract content that would be present in the tablets after a standardized production process.

### Determination of the *X*. *americana* tablets dissolution profile

#### Development of the quantification method

In order to provide a simple, fast, and low-cost method for extract quantification in the tablets, a spectrophotometric quantification method in the UV-visible region was developed for the dissolution profile study of the tablets. Solutions of the extract, dissolved in phosphate buffer solution (pH = 6.8), were analyzed. The spectrophotometric scans were performed in a Lambda 950 spectrophotometer (Perkin Elmer), between the wavelengths of 1100–190 nm.

The quantification method was validated by analyzing the following parameters: specificity, linearity, repeatability, intermediate precision, accuracy, detection and quantification limits, and robustness [10]

#### Specificity

Specificity of the quantification method was determined by analyzing the influence of the formulation of pharmaceutical excipients on the extract absorption bands. For this parameter, spectrophotometric readings of the extract, excipient matrix, and tablet formulation solutions were performed in the UV-visible region and were evaluated for the occurrence of band displacements or overlaps.

#### Linearity

Method linearity was analyzed by constructing calibration curves of the extract in increasing concentrations. Three calibration curves were analyzed for the determination coefficients (R^2^), residues distribution, lack of fit test, and regression significance test, using F values for 5% significance.

#### Repeatability and intermediate precision

The repeatability was evaluated by analyzing solutions of the extract at three different concentration levels: low, medium, and high (50, 175, and 300 μg mL^-1^, respectively). For the intermediate precision, these samples were analyzed on three different days. All samples were analyzed in triplicate.

#### Accuracy

Accuracy was evaluated by comparing the concentrations determined in extract solutions at three different concentration levels (50, 175, and 300 μg mL^-1^) with their theoric concentration values.

#### Detection and quantification limits

Detection and quantification limits were determined from the standard deviation of the spectrum baseline noise.

#### Robustness

The robustness was determined by applying deliberate changes in analysis conditions (different analysts and reading temperatures of the samples).

#### Determination of the dissolution profile

A dissolution equipment model 299 (Ethik Technology) was used. Six tablets were dissolved in 900 ml of phosphate buffer solution (pH 6.8), using the paddle apparatus at 37 °C and rotation of 75 rpm. During the dissolution test, 3 mL aliquots were taken at 5, 10, 15, 20, 25, 30, 45, and 60 minutes. These were subjected to spectrophotometric reading, using the quantification method developed. The robustness of the dissolution method was analyzed by deliberate variations in the test conditions; these variations were in the agitation and in the medium temperature.

## Results and discussion

### Fingerprint of the *X*. *americana*

**Fig 1:**
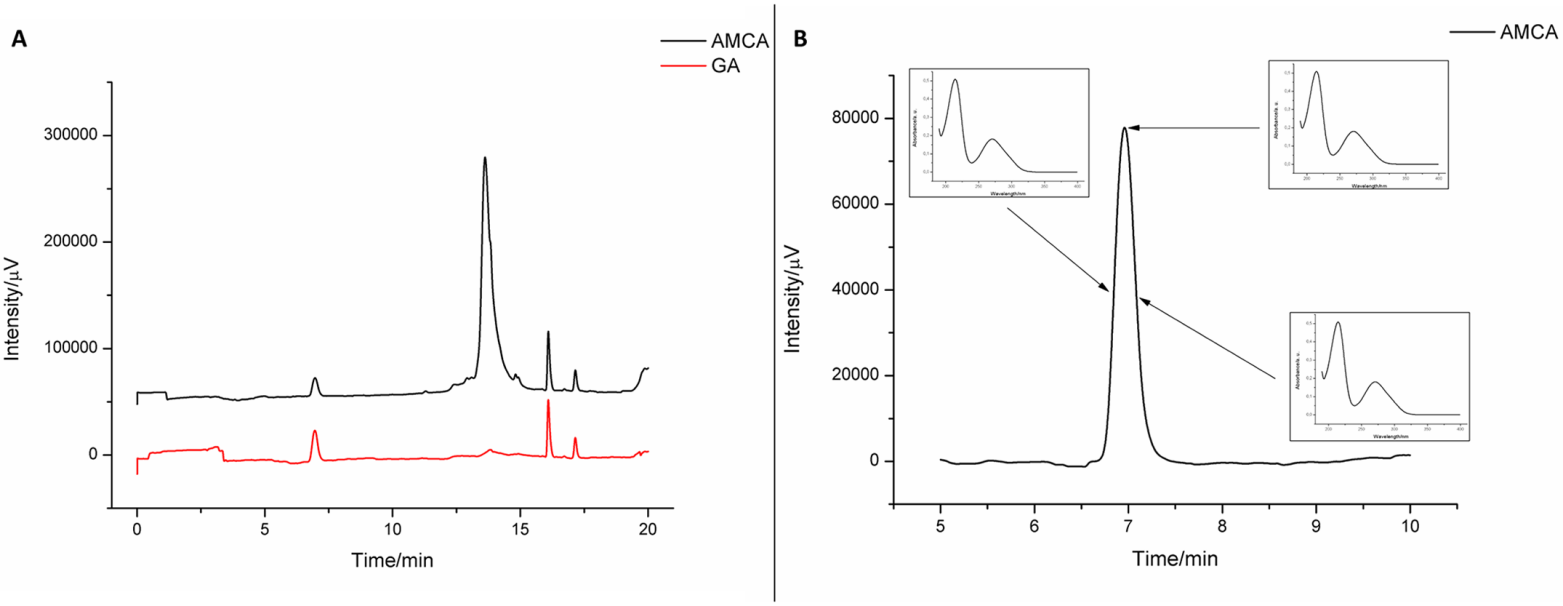
Specificity analysis: (A) Comparison of gallic acid (GA) peaks in the extract and in the chromatographic standard; (B) UV-Vis spectra of medium and maximum heights of the gallic acid peak in the extract AMCA.

Among the chromatographic standards used, gallic acid was the one which corresponded with peak retention time. The gallic acid from the extract was successfully separated by the chromatographic method and presented at the retention time of 6.90 min. The other secondary metabolites were detected at retention times above 12.50 min. These were co-eluted, probably due to the increased methanol concentration in the mobile phase; increased concentration can carry hydrophilic substances such as polyphenols, flavonoids, and tannins, which are present in the extract, according to the quantifications and results presented in Table 1. The chromatograms of the extract and gallic acid are compared in Fig 1A.

**Table 1:** Concentrations of secondary metabolites in the *X*. *americana* extract.

Secondary metabolites	Content (mg/g)
Total polyphenols	160.08 ± 1.15^1^
Total flavonoids	11.26 ± 0.06^2^
Condensed tannins	94.75 ± 0.51^3^

^1^Gallic acid equivalent (GAE),

^2^Quercetin equivalent (QE),

^3^Catechin equivalent (CE).

**Fig 2:**
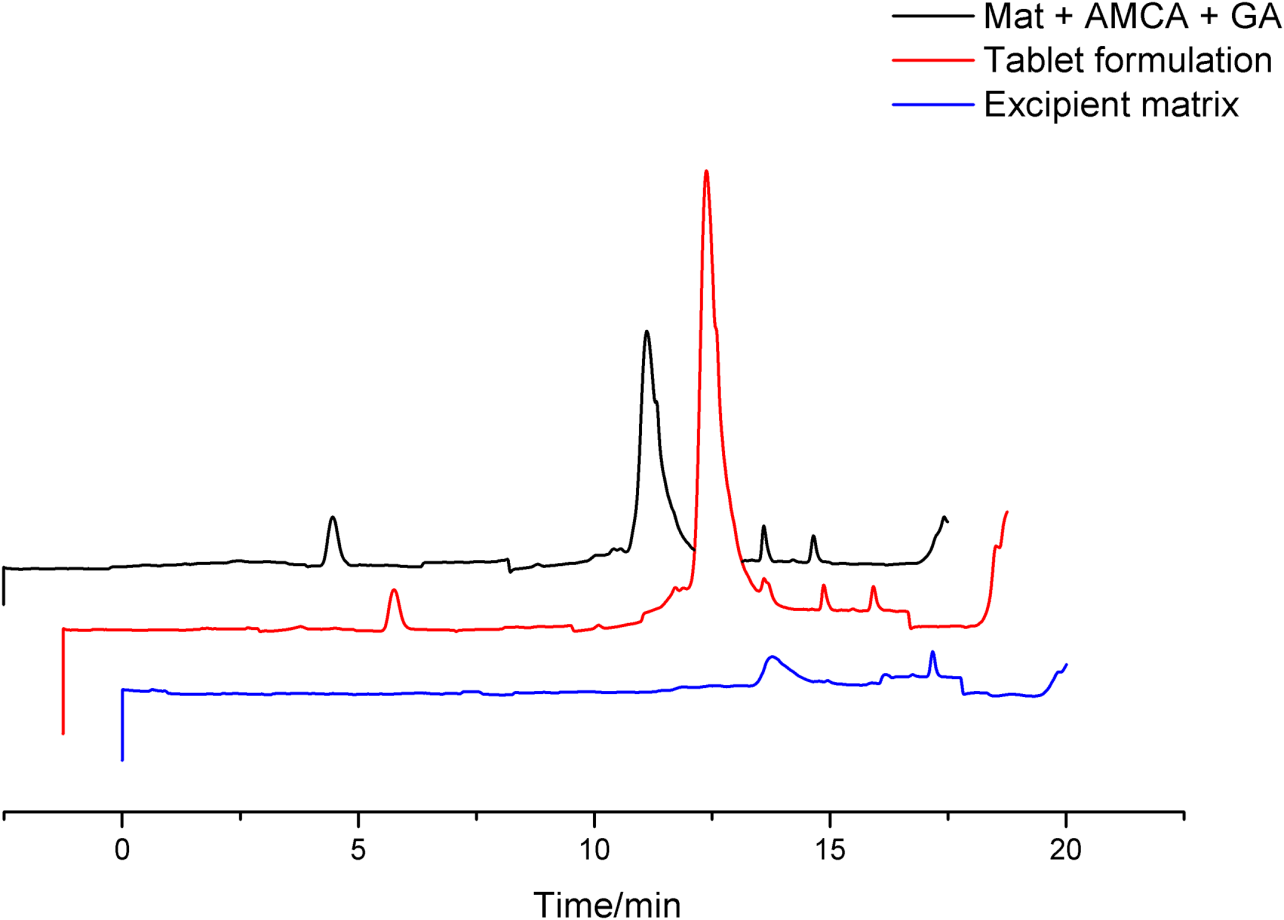
Specificity analysis: Comparison of chromatograms of the tablet formulation, excipient matrix and mitxture of extract, excipients, and gallic acid.

Fig 1B also shows the specificity analysis of the chromatographic peak for gallic acid present in the extract. Three UV-Vis spectra of the medium and the maximum peak height value, respectively, were compared, and their calculated similarity was 98.90% (Table 2). This result shows high similarity of spectra, indicating high separation efficiency. It was found that the excipient matrix in the tablet formulation does not interfere in the separation of gallic acid, since co-elution of other substances does not occur and gallic acid is detected at the same retention time. Therefore, this separation method was considered effective for the chemical marker quantification of the extract (Fig 2).

**Table 2:** Validation parameters for the chromatographic quantification method of the *X*. *americana* extract.

**Repeatability (CV%)**				
Level	Day 1	Day 2		Day 3
Low	3.58	3.71		0.60
Medium	8.79	0.79		2.29
High	2.25	1.20		0.18
**Intermediate precision (CV%)**				
Level				
Low	11.10	-		-
Medium	6.60	-		-
High	8.48	-		-
**Accuracy (%)**				
Level		Intraday		Interday
Low	Day 1	95.81		102.66
	Day 2	102.17		
	Day 3	110.00		
Medium	Day 1	110.01		102.92
	Day 2	102.28		
	Day 3	96.46		
High	Day 1	86.25		95.07
	Day 2	102.06		
	Day 3	96.89		
**Detection and quantification limits (μg mL**^**-1**^**)**				
D. limit	0.03	-		-
Q. limit	0.10	-		-
**Specificity**				
Peak purity (%)	98.90	-		-
Retention time (min)	6.91	-		-
**Linearity**				
R^2^ coefficient	0.9978	-		-
Line equation	y = 31233x + 22.53			-
(MQ_faj_/MQ_ep_)/F	0.479/3.71		-	-
(MQ_reg_/MQ_r_)/F	1294.990/4.67		-	-
Residue analysis	Homoscedastic residue distribution			

MQ_faj_ = Lack of fit quadratic mean

MQ_ep_ = Pure error quadratic mean

MQ_reg_ = Model adjustment quadratic mean

MQ_r_ = Residual quadratic mean

**Fig 3:**
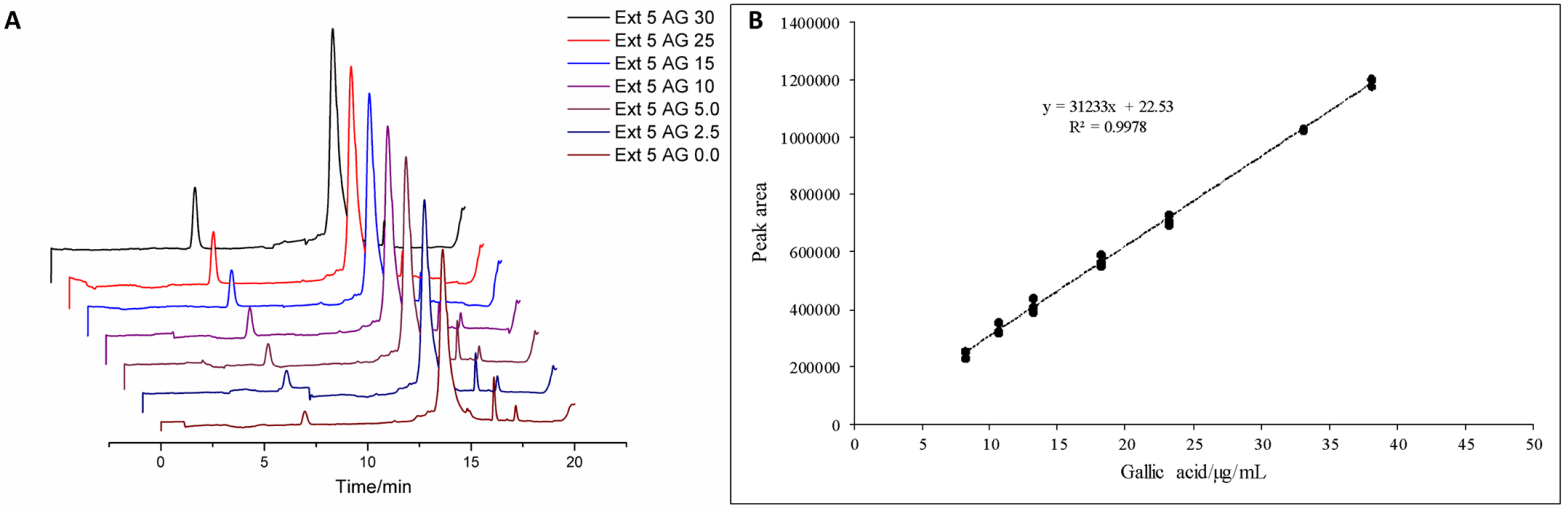
Linearity analysis: (A) Calibration curve chromatograms; (B) Calibration curve data.

For the gallic acid quantification method, a linear calibration model was established by applying standard addition at increasing concentrations in the extract solutions. As shown in Fig 3, the calibration curve was linear between the addition concentrations of 0 to 30 μg mL^-1^. As a chemical marker, this substance was taken into account as a parameter for quality control, to calculate the final amount of the vegetal extract in the finished tablets [4].

The results obtained for repeatability and intermediate precision show that the gallic acid quantification method gives concordant results, even in the presence of pharmaceutical excipients. This information is important, since these compounds are often interfering elements and should be considered in the analysis of pharmaceutical formulations in the industry [13]. The coefficients of variation (CV%) found were all below 10%, as shown in Table 2. For these parameters, these values indicate a satisfactory accuracy degree, according to studies carried out by Kumar et al. [14] and Kartini [15]; the values may be associated with the complexity of the matrix in which the chemical marker was quantified, which includes a wide variety of phytochemical compounds and pharmaceutical excipients.

Accuracy analysis was performed using three concentrations (2.50, 15.00, and 25.00 μg mL^-1^) of the standard, added to extract solutions at 5.00 mg mL^-1^, in order to consider the entire range of calibration curve.

The results of the intra-day and inter-day accuracy analysis, presented in Table 2, ranged from 86.25 to 110.01%, which is within the RDC-recommended 899/2003 of ANVISA [16]—a variation lower than 15%—and can be considered acceptable for the quantification of chemical markers in plants.

The detection and quantification limits, calculated from the standard deviation of the chromatogram baseline, are presented in Table 2; these are 0.03 and 0.10 μg mL^-1^, respectively. These values are below the minimum concentration of gallic acid quantified in the extract solutions (8.08 μg mL^-1^), allowing chemical marker analysis at the concentrations studied.

The efficient validation of the chromatographic method made it possible to quantify the gallic acid content of the extract, which was equal to 1.61 mg g^-1^.

#### Formulation development

Three formulations, presented in Table 3, were produced by using the excipients that showed compatibility with the dry extract; these were analyzed in the development studies, in order to choose the one most suitable for compression. The extract was also analyzed without excipients.

**Table 3:** Percentual component concentration of the formulations proposed for the tablet.

Component*	Formulation 1 (%)	Formulation 2 (%)	Formulation 3 (%)
MC 102	35.50	33.00	41.00
PVP K-30	2.50	5.00	1.00
TAL	10.00	10.00	10.00
CSD	1.00	1.00	1.00
CRO	5.00	5.00	1.00
AMCA	46.00	46.00	46.00

*MC 102: microcrystalline cellulose PH 102; PVP K-30: polyvinylpyrrolidone K-30; TAL: talc; CSD: colloidal silicone dioxide; CRO: sodium croscarmellose; AMCA: *X*. *americana* dry extract.

As expected, the extract showed undesirable flow properties [6] (Table 4), which may be due to its amorphous and hygroscopic characteristics (the dry extract presented a moisture content of 7.91% [11]), even in the presence of a drying adjuvant. This shows the need to incorporate lubricants and glidants into the formulation, in order to make the plant extract suitable for the direct compression method.

**Table 4:** Characterization data of the powders in the formulation study.

	AMCA	Form. 1*	Form. 2**	Form. 3***
Bulk density (g mL^-1^)	0.32	0.41	0.41	0.36
Compaction density (g mL^-1^)	0.55	0.52	0.58	0.58
**Hausner ratio**	1.70	1.25	1.39	1.60
Carr index (%)	41.30	20.31	28.35	37.80
Densification index (mL)	3.66	4.00	5.33	5.66
Repose angle (°)	-	< 25	< 25	< 25
Flow time (s)	∞	0.91	0.78	0.62

*Form. 1: formulation 1;

**Form. 2: formulation 2;

***Form. 3: formulation 3.

Formulations 1, 2, and 3 presented excellent repose angles (Table 4), all below 25° (excellent flow), which demonstrated the improvement in flow characteristics with the added excipients. However, the flow times were shorter, with low concentrations of polymers, as in formulation 3.

Indirect techniques for determining the technological properties of powders include the Hausner ratio and the Carr index; these are also taken into account in solid formulation studies [17]. However, the results obtained for these coefficients indicated intermediate properties for all the samples (Table 4).

Hausner ratio (HR) is an indirect measure of the powder flow ease; according to its classification, the analyzed powders had poor (AMCA and formulation 3) and moderately good (formulations 1 and 2) flow properties, which conflicts with the information given by the repose angle test [18].

The Carr index (CI) indirectly measures the suitability of a powder for compression, regarding its interparticular interactions [19]. The results classify the formulations powders as very, very poor (AMCA), reasonable (formulation 1), poor (formulation 2), and very poor (formulation 3). These results are probably related to the cohesion degree between the polymer particles in the densification process.

With the reduction in the PVP K-30 and sodium croscarmellose concentrations from formulation 1 to formulation 3, the densification index is increased (Table 4), which shows that the formulation exhibits greater particle reorganization property. This property allows it to form more compact, uniform, and resistant tablets after compression. Therefore, formulation 3 was chosen as the most suitable for direct compression, according to the direct flow measures.

### Development and standardization of *X*. *americana* tablets

**Fig 4:**
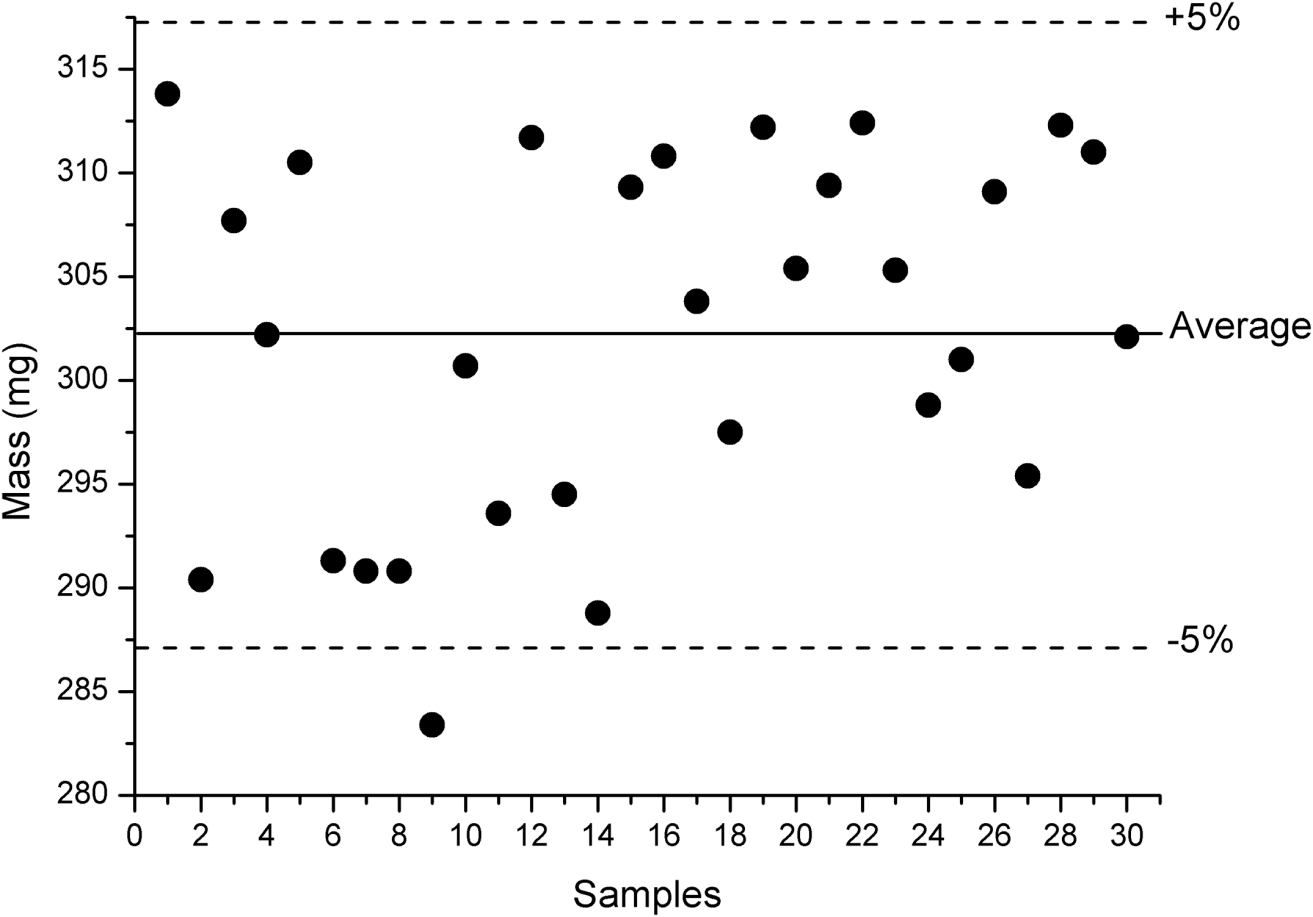
Data from the average weight determination.

Thirty tablets were taken for the average weight determination, comprising the entire compression process. The measurements are shown in Fig 4. The calculated average weight of the tablets was equal to 302.20 mg (Table 5), with measurements varying from 283.40 to 313.80 mg. This first value is below the 5% deviation limit, which is recommended by The United States Pharmacopeia [12]; however, since only one measurement was below the limit, the result of the average weight determination was considered satisfactory, with a good distribution of the formulation in each compression cycle. This also indicates that desirable flow properties were obtained in formulation 03.

**Table 5:** Data from the tablet batch characterization.

Parameter	Result	Specification
Average weight (mg)	302.20 ± 8.75	287.09 to 317.31
Hardness (N)	45.15 ± 18.87	40.00 to 60.00
Friability (%)	0.34	< 1.50
Disintegration time (min)	6.11 ± 2.03	< 30

The produced tablets had an average hardness of 45.15 N (Table 5). Afifi and Ahmadeen [19] point out that the minimum hardness to ensure the physical stability of tablets is 40.00 N, which shows that the obtained batch fulfills the specification for hardness. In addition, the friability calculated for the tablets was equal to 0.34% (Table 5), which is also in accordance with the maximum limit of 1.50% that is recommended by the The United States Pharmacopeia [12].

The mean disintegration time for the analyzed tablets was equal to 06 min 08 s (Table 5), for the purified water medium at 37 °C. This disintegration time is desirable for providing the beginning of the dissolution process over a short range of time. The The United States Pharmacopeia [12] recommends that the maximum disintegration time of immediate-release tablets is 30 min.

The extract content determined in the tablets by chromatographic method (Table 6) varied between 92.78 and 99.35%, with a variation of less than 10%, which can be considered a discrete content variation [12].

**Table 6:** Data from the content uniformity analysis by HPLC.

	Extract content (mg)		Label amount (%)
Sample	Measured	Expected	
1	207.19	222.92	92.94
2	208.46		93.51
3	221.49		99.35
4	219.00		98.24
5	206.83		92.78

### Determination of the *X*. *americana* tablets dissolution profile

**Fig 5:**
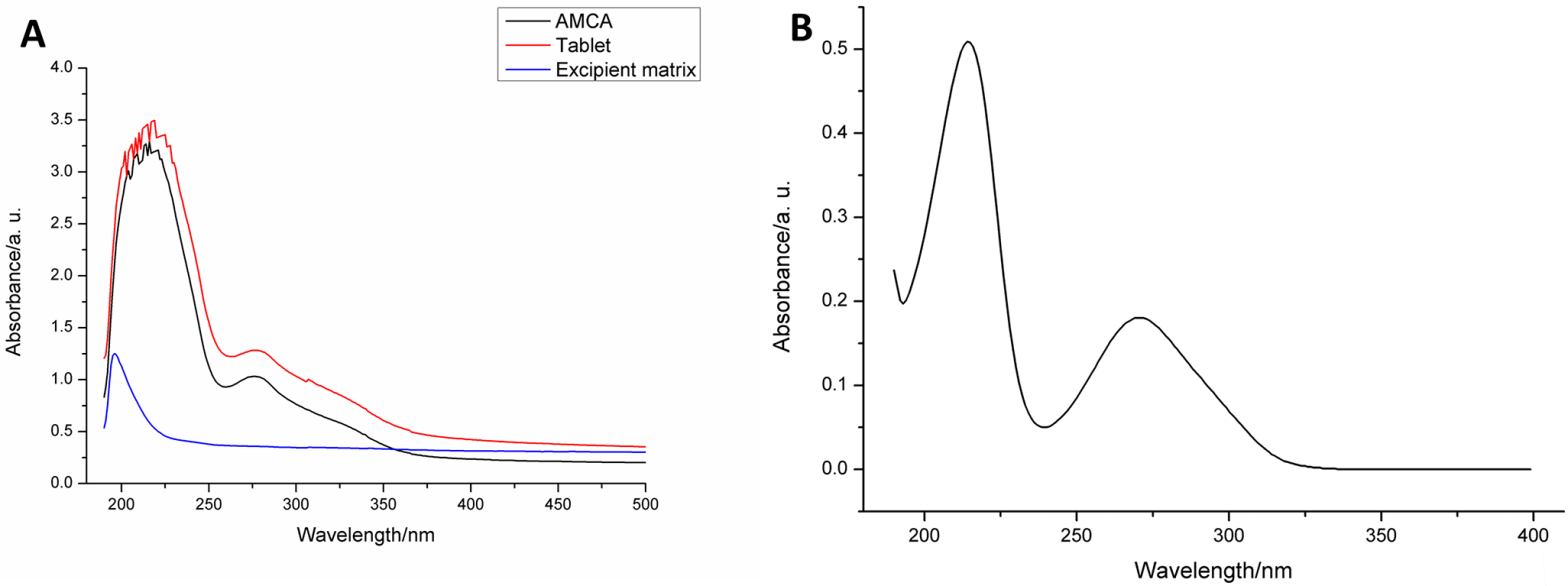
Specificity analysis: (A) UV-Vis spectra of the analyzed samples; (B) UV-Vis spectrum of the chemical marker (gallic acid).

The spectrophotometric scanning of the extract presented an absorbance band in the UV region, with a peak at 276 nm (Fig 5A). This wavelength was taken as reference for the spectrophotometric extract quantification.

Fig 5B also presents the specificity analysis of the spectrophotometric quantification method. It was observed that the absorbance band with peak at 276 nm is not influenced by the presence of the tablet excipient matrix, and that it, in turn, does not present absorbance in that region. Thus, it can be affirmed that the developed method is specific and can be used to detect the extract in the UV region.

**Fig 6:**
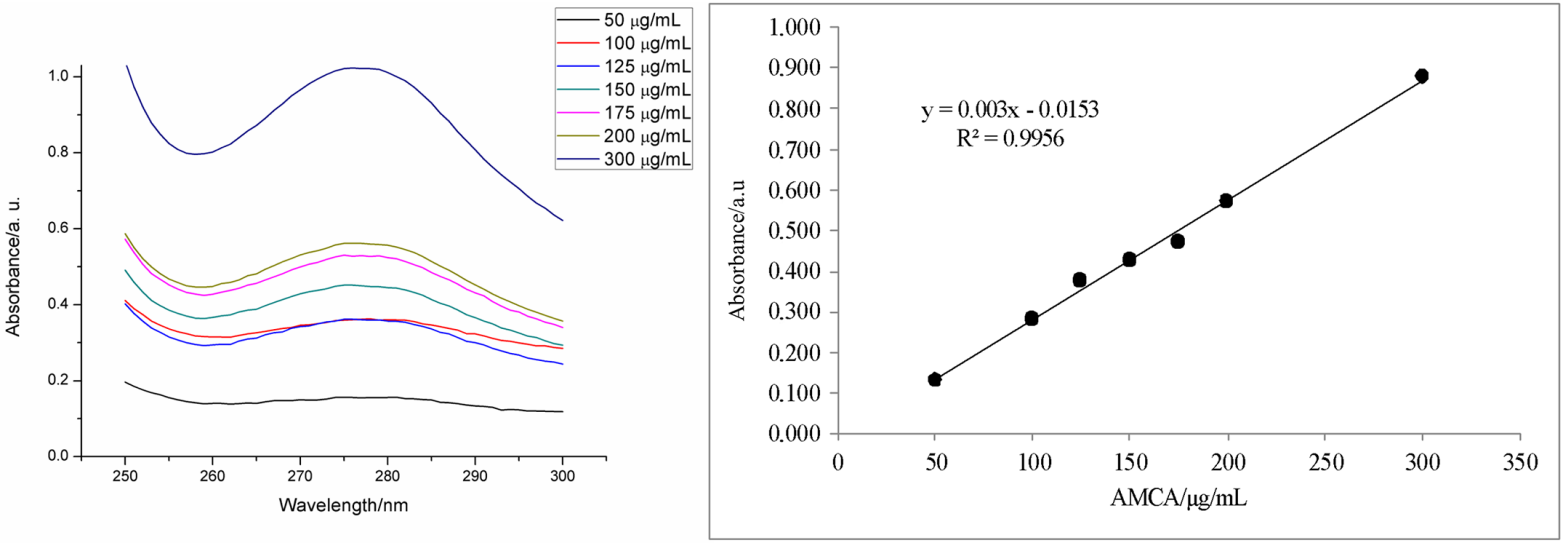
Linearity analysis: (A) spectra of the calibration curve; (B) calibration curve data.

The spectra obtained in this range (Fig 6) showed quantitative differentiation of extract concentration; consequently, calibration curves were obtained with satisfactory linear adjustment, with a determination coefficient equal to 0.9956. The other data concerning the method linearity are shown in Table 7.

**Table 7:** Validation parameters for the spectrophotometric quantification method of the extract in tablet.

**Repeatability (CV%)**					
Level	Day 1	Day 2			Day 3
Low	2.40	5.97			9.99
Medium	6.18	1.36			1.06
High	4.34	5.06			3.00
**Intermediate precision (CV%)**					
Level					
Low	5.23	-			-
Medium	9.45	-			-
High	8.94	-			-
**Accuracy (%)**					
Level		Intraday			Interday
Low	Day 1	111.20			104.33
	Day 2	95.56			
	Day 3	106.22			
Medium	Day 1	111.80			101.47
	Day 2	92.27			
	Day 3	100.34			
High	Day 1	98.82			93.27
	Day 2	86.58			
	Day 3	94.41			
**Detection and quantification limits (μg/mL)**					
D. limit	12.89	-			-
Q. limit	39.08	-			-
**Specificity**					
Absorption peak (nm)	276				-
**Linearity**					
R^2^ coefficient	0.9956	-			-
Line equation	y = 0.003x – 0.0153				-
(MQ_faj_/MQ_ep_)/F	0.297/2.96		-		-
(MQ_reg_/MQ_r_)/F	319.616/4.38		-		-
Residue analysis	Homoscedastic residue distribution				
**Robustness (CV%)**					
Analyst 1	Low			8.77	
	Medium			12.60	
	High			12.95	
Analyst 2	Low			4.26	
	Medium			3.16	
	High			8.78	
30 °C	Low			3.55	
	Medium			13.57	
	High			17.34*	
40 °C	Low			1.54	
	Medium			18.53*	
	High			18.96*	

* = Variation considered significant (higher than 15.00%)

MQ_faj_ = Lack of fit quadratic mean

MQ_ep_ = Pure error quadratic mean

MQ_reg_ = Model adjustment quadratic mean

MQ_r_ = Residual quadratic mean

Table 7 also shows data on repeatability, intermediate precision, and accuracy of the method. For the first two, the variation coefficient results (less than 10%) and the accuracy results (variation lower than 15%) indicate a high efficiency of the method. This was able to quantify the concentration of the extract without need for sample pre-processing, and to provide concordant results on every day of analysis [14,15].

The detection and quantification limits (Table 7) obtained for the wavelength 276 nm were considered satisfactory, since they correspond to concentrations below the linear calibration model. These results indicate that the characteristic baseline noise of this equipment does not interfere in determining solutions at the concentrations within the curve.

The method was considered robust against the analyst variation applied to the test, showing variations smaller than 15% (Table 7) in the reading of the prepared samples in all three levels analyzed. However, the variation of the sample reading temperature was shown to be an interference parameter for this method, with variations higher than 15% at temperatures of 30 and 40 °C at the medium and high levels of analysis (Table 7). This variation may be due to the possible degradation of thermosensitive components of the extract, which may occur with increasing temperature; it may also be due to changes in the ionization profile of the polyphenolic compounds present, which can cause changes in the absorption intensity or band displacements in the UV region.

**Fig 7:**
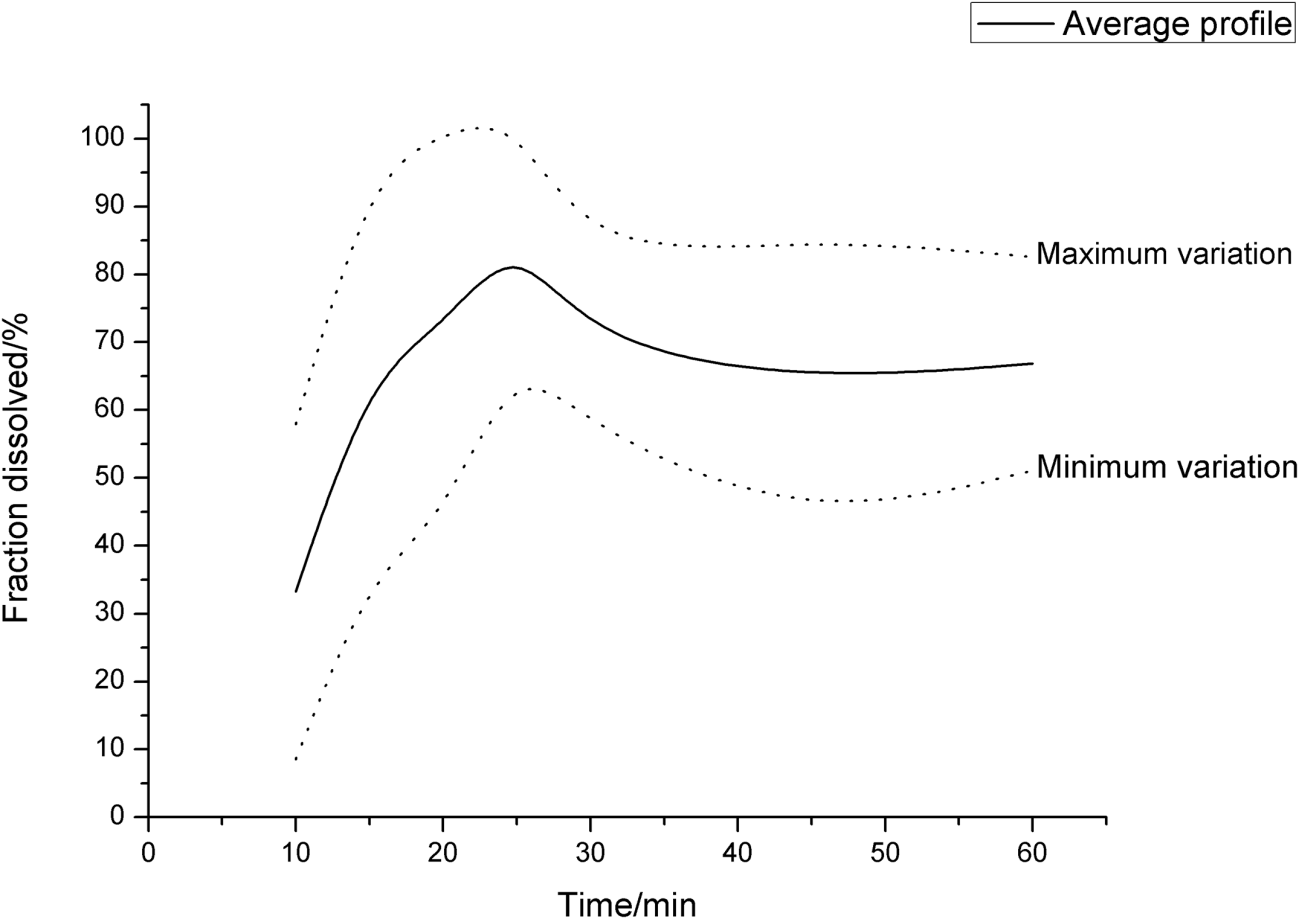
Average dissolution profile of the tablets with minimum and maximum variations.

The average dissolution profiles obtained for the evaluated tablets—with their minimum and maximum variation—are shown in Fig 7. In the profile region between zero and 30 minutes, a phase of rapid dissolution is observed in which the concentrations vary from 33.23% at 10 min to a maximum of 86.00% at 25 min.

The significant fraction dissolved in the first point of the profile, and the rapid dissolution up to the time of 25 min, are probably related to the presence of croscarmellose sodium in the formulation, which provides a rapid disintegration of the tablet and thereby initiates the process of dissolution, rapidly and uniformly, throughout the tablet [20].

At 5 min, the presence of the extract dissolved in the medium was not detected in any of the analyzed tablets. This finding is related to the time required for the beginning of the disintegration process of the tablet—which in all samples was greater than 5 and less than 10 min—and corroborates with the result found in the disintegration time test. During the first 5 min, the tablet is probably still in the water-absorption phase from the medium by the disintegrant, an influx of solvent required for disintegration to occur. After this step, the disintegration process occurs rapidly.

After the time of 25 min, the average dissolution profile shows a drop in the dissolved fraction, from 86.00 to 69.48% in 30 min, and then stabilizes between 64.27 and 66.84% at 45 min. This behavior is usually uncommon in dissolution studies; it indicates the possible formation of a supersaturated extract system in the dissolution medium at the time of 25 min, with subsequent precipitation of the dissolved excess at this time, which causes the drop in the dissolved fraction observed at 30 min.

The formation of supersaturated systems during tablet dissolution is reported in the literature mainly for amorphous APIs (as is the case of *X*. *americana* extract), due to its high solubility and due to the absence of a crystalline structure in its particles. The dissolution is accelerated both by the presence of a "superdisintegrant" like croscarmellose, as well as by the presence of PVP K-30 as a binder in the formulation [21].

PVP K-30, dispersed in the dissolution medium, would have the ability to aggregate dissolved compounds of the extract through interactions mediated by electrostatic attractions. The formation of these soluble complexes in the medium would favor the extract dissolution in greater extent, until the formation of a supersaturated system. PVP is also reported to have the property of increasing the dissolved fraction that stabilizes in the medium after precipitation of excess API [22,23]. This indicates that subsequent changes in the proportion of PVP K-30 in the formulation could be made, in order to increase the dissolved fraction of the extract after the time of 30 min.

A robustness evaluation of the dissolution method was performed, and the results are presented in Table 8. The data show that the variation in the medium temperature to 32 °C did not affect the dissolution profile of the tablets; however, the increase in temperature to 42 °C provided a significant increase in the fraction dissolved in the first 20 minutes of the assay. The increase in temperature was thus considered a critical factor of variation in the assay, probably related to its influence on tablet disintegration.

**Table 8:** Variation coefficients regarding the robustness evaluation of the dissolution test.

Condition	Time (min)	Variation coefficient (CV%)
32 °C; 75 rpm	05	12.48
	10	0.07
	15	2.10
	20	11.01
	25	12.01
	30	6.13
	45	14.27
	60	1.25
42 °C; 75 rpm	05	99.46*
	10	81.39*
	15	53.58*
	20	31.08*
	25	3.82
	30	12.60
	45	13.18
	60	3.08
37 °C; 50 rpm	05	81.96*
	10	78.05*
	15	49.66*
	20	20.73*
	25	2.01
	30	9.64
	45	12.59
	60	9.76
37 °C; 100 rpm	05	44.39*
	10	62.50*
	15	49.04*
	20	9.27
	25	2.89
	30	8.26
	45	0.24
	60	9.10

* = Variation considered significant (higher than 15.00%)

Changes in rotation to both 50 and 100 rpm were shown to be variation factors in the test up to 20 min. This variation is probably related to changes in the mechanical force that intensifies the disintegration process of the tablet.

## Conclusion

The study developed a high-content tablet formulation from *X*. *americana* extract, which was suitable for production by direct compression. For this procedure, the use of adjuvants was fundamental in improving the extract’s flow properties.

The dissolution profile of the tablets presented the possibility of forming a supersaturated system during the dissolution test. This phenomenon, although unusual in studies of this nature, relates to the solid state properties of the extract and to the composition of the formulation itself.

The determination and quantification of the chemical marker in the extract were an important step in development, not only to provide knowledge of its composition, but mainly to provide an important quality parameter of the finished product, which is fundamental to guaranteeing the therapeutic efficacy of the product.

## Supporting information

S1 File Certificate of writing correction. (PDF)

S2 File Electronic form of the National System of Genetic Heritage and Associated Traditional Knowledge (SISGEN). (PDF)

S3 File Data obtained for the HPLC quantification method. (XLSX)

S4 File Data obtained for the UV/VIS quantification method. (XLSX)

S5 File Data obtained in the characterization of the formulations and tablets. (XLSX)

S6 File Data obtained for the tablet dissolution method. (XLSX)

## References

[1] Cartaxo SL. Souza MMA. Albuquerque UP. Medicinal plants with bioprospecting potential used in semi-arid northeastern Brazil. J Ethnopharmacol. 2010; 131: 326–342. doi: 10.1016/j.jep.2010.07.003 20621178 10.1016/j.jep.2010.07.003

[2] Silva MSP. Brandão DO. Chaves TP. Formiga Filho AL. Costa EMMD. Santos VL. et al Study bioprospecting of medicinal plant extracts of the semiarid northeast: contribution to the control of oral microorganisms. J Evid Based Complementary Altern Med. 2012 doi: 10.1155/2012/681207 22719786 10.1155/2012/681207 PMC3375581

[3] Silva KMA. Chaves TP. Santos RL. Brandão DO. Fernandes FHA. FJD LR Júnior. et al Modulation of the erythromycin resistance in Staphylococcus aureus by ethanolic extracts of *Ximenia americana* L and *Schinopsis brasiliensi*s Engl. *B*. *Latinoam*. Caribe Pl. 2015; 14:92–98.

[4] Rasheed NMA. Nagaiah K. Goud PR. Sharma VUM. Chemical marker compounds and their essential role in quality control of herbal medicines. Ann. Phytomed. 2012;1: 1–8.

[5] Fernandes HA, Santana CS, Silva PCD, Simões MOS, Kaneko TM, Medeiros ACD. Development of a sunscreen by thermal compatibility study using *Schinopsis brasiliensis* Engler extract as preservative. J Therm Anal Calorim. 2017; doi: 10.1007/s10973-017-6437-7

[6] Gallo L, Ramírez-Rigo MV, Wilson E, Piña J, Allemandi D, Bucalá V. Spray-Dried Cascara Sagrada extract for direct compression: tablet formulation and a simple HPLC method for tablet performance evaluation. Int J Res Pharm Biomed. Sci. 2013; 4: 1360–1370.

[7] Fernandes FHA, Almeida VE, Medeiros FD, Silva PCD, Simões MOS, Véras G. et al Evaluation of compatibility between *Schinopsis brasiliensis* Engler extract and pharmaceutical excipients using analytical techniques associated with chemometric tools. J Therm Anal Calorim. 2016; 123: 2531–2542. doi: 10.1007/s10973-016-5241-0

[8] Chaves TP, Fernandes FHA, Santana CP, Santos JS, Medeiros FD, Felismino DC. et al Evaluation of the interaction between the *Poincianella pyramidalis* (Tul.) LP Queiroz extract and antimicrobials using biological and analytical models. PLoS ONE. 2016; doi: 10.1371/journal.pone.0155532 27192209 10.1371/journal.pone.0155532 PMC4871567

[9] Makkar HPS, Becker K. Vanillin-HCl method for condensed tannins: Effect of organic solvents used for extraction of tannins. J Chem Ecol. 1993; 31: 613–621. doi: 10.1007/BF00984996 24249005 10.1007/BF00984996

[10] International conference on harmonization of technical requirements for registration of pharmaceuticals for human use—ICH. Validation of analytical procedures: text and methodology Q2 (R1). 2005; 1–13.

[11] Santana CP, Fernandes FHA, Brandão DO, Silva PCD, Correia LP, Nóbrega FP, et al Véras G. Medeiros ACD. Compatibility study of dry extract of *Ximenia americana* L. and pharmaceutical excipients used in solid state. J Therm Anal Calorim. 2017; doi: 10.1007/s10973-017-6764-8

[12] The United States Pharmacopeia, 30 ed/ National Formulary 25. The United States Pharmacopeial Convention (USP), Rockville. 2013.

[13] Fereja TH, Seifu MF, Mola TY. UV-Visible Spectrophotometric Method Development and Quantification of Ciprofloxaciline in Tablets Dosage Form. J Pharm Pharmacol. 2015; 2: 1–8.

[14] Kumar N, Tharatha S, Chaiyasut C. Development and validation of simple isocratic high performance liquid chromatography-ultraviolet (HPLC-UV) method for determination of safflower yellow in *Carthamus tinctorius* L.-loaded nanostructured lipid carriers (NLC). Afr J Pharm Pharmacol. 2011; 5: 2335–2341.

[15] Kartini A. Chromatographic fingerprinting and clustering of *Plantago major* L. from different areas in Indonesia. Asian J Pharm Clin Res. 2012; 5: 191–195.

[16] BRASIL, Ministério da Saúde. Agência Nacional de Vigilância Sanitária (ANVISA). Resolução RE n° 899, de 29 de maio de 2003. Diário Oficial da União, Brasília. 2003.

[17] Bernatoniene J, Petkeviciute Z, Kalveniene Z, Masteikova R, Draksiene G, Muselik J, et al The investigation of phenolic compounds and technological properties of *Leonurus*, *Crataegus* and Ginkgo extracts. J Med Plant Res. 2010; 4: 925–931.

[18] Gupta R, Sharma P, Garg A, Soni A, Sahu A, Rai S, et al Formulation and Evaluation of Herbal Effervescent Granules Incorporated with *Calliandra Haematocephala* Leaves Extract. Indo Am J Pharma Res. 2013; 3: 4366–4371.

[19] Afifi SA, Ahmadeen S. A comparative study for evaluation of different brands of metformin hydrochloride 500 mg tablets marketed in Saudi Arabia. Life Sci J. 2012; 9: 4260–4266.

[20] Medeiros ACD, Correia LP, Simões MOS, Macêdo RO. Technogical quality determination of pharmaceutical disintegrant by DSC cooling and DSC photovisual. J Therm Anal Calorim. 2007: 88: 311–315. doi: 10.1007/s10973-006-8005-4

[21] Van Drooge DJ, Hinrichs WLJ, Frijlink HW. Anomalous dissolution behaviour of tablets prepared from sugar glass-based solid dispersions. J Control Release. 2004; 97: 441–452. doi: 10.1016/j.jconrel.2004.03.018 15212876 10.1016/j.jconrel.2004.03.018

[22] Abu-Diak OA, Jones DS, Andrews G. P. An investigation into the dissolution properties of celecoxib melt extrudates: understanding the role of polymer type and concentration in stabilizing supersaturated drug concentrations. Mol Pharm. 2011; 4: 1362–1371. doi: 10.1021/mp200157b 21696180 10.1021/mp200157b

[23] Qian F, Wang J, Hartley R, Tao J, Haddadin R, Mathias N, et al Solution behavior of PVP-VA and HPMC-AS-based amorphous solid dispersions and their bioavailability implications. Pharm Res. 2012; 29: 2766–2776. doi: 10.1007/s11095-012-0695-7 22315020 10.1007/s11095-012-0695-7

