# Supplementary material for: Dissolution and uniformity of content of tablets developed with extract of Ximenia americana L
Source: PLoS One. 2018 May 24;13(5):e0197323. doi: 10.1371/journal.pone.0197323 (PMC5993115; doi:10.1371/journal.pone.0197323)
Supplement: S1 File — (PDF) [file pone.0197323.s001.pdf]

EditMyEnglish

English Editing Certificate

This document certifies that the manuscript listed below was edited for proper English language, grammar, punctuation, and spelling by the expert staff at EditMyEnglish.

Manuscript Title:

Dissolution and uniformity of content of tablets developed with extract of Ximenia americana L.

Authors:

Cleildo P. Santana, Francinalva D. de Medeiros, Lidiane P. Correia, Paulo Henrique G. D. Diniz, Germano Véras, Ana Cláudia D. Medeiros

Certificate Verification Key:

244-476-271-351-604

Project Number:

84043

This certificate may be verified by emailing info@editmyenglish.com. Documents receiving this certificate should be prepared for publication. However, please note that the author has the ability to accept or reject our suggestions for changes and can make changes after the editing process is complete, all of which can adversely affect the quality of the text after the editing process.

Edit My English
