# Supplementary material for: Dissolution and uniformity of content of tablets developed with extract of Ximenia americana L
Source: PLoS One. 2018 May 24;13(5):e0197323. doi: 10.1371/journal.pone.0197323 (PMC5993115; doi:10.1371/journal.pone.0197323)
Supplement: S2 File — (PDF) [file pone.0197323.s002.pdf]

SisGen

Sistema Nacional de Gestão do Patrimônio Genético e do Conhecimento Tradicional Associado

(../paginas/home.aspx)

Tempo Restante: 29:42

Cadastro de Atividade de Acesso

Tipo de Usuário:

Independente *

Responsável pelo cadastro

Adicionar + *

CPF	Habilitado		
92956467468	Sim		

Objeto do Acesso:

Patrimônio Genético *

O acesso foi realizado antes de 17/11/2015 ou obteve autorização de acesso antes de 17/11/2015?

Sim *

Tem autorização de acesso?

Não – Sem solicitação em tramitação *

Finalidade do Acesso: *

- [x] **Pesquisa Científica**
- [ ] **Bioprospecção**
- [ ] **Desenvolvimento Tecnológico**

As atividades objeto deste cadastro são baseadas em outras atividades de acesso realizadas anteriormente?

Não *

Este cadastro está vinculado a cadastro anterior de remessa?

Não *

Patrimônio Genético

Título da Atividade:

Desenvolvimento de comprimidos com atividade antimicrobiana a partir de extrato de Ximenia americana *

Título da Atividade em inglês:

Development of tablets with antimicrobial activity from extract of Ximenia americana L.

Resumo da atividade (incluindo objetivos e resultados esperados ou obtidos, conforme o caso)

O uso de plantas para fins medicinais é uma tradição em diversas culturas, em todo o mundo, desde épocas remotas. Ainda hoje, o conhecimento popular fornece informações sobre propriedades medicinais de inúmeras espécies, efeitos colaterais e adversos, e instruções para seu cultivo e preparo para uso no tratamento de enfermidades. Embora o conhecimento da medicina tradicional não seja mais tão intensamente difundido, a tradição do uso de plantas medicinais persiste na população, que frequentemente faz uso de medicamentos fitoterápicos, tradicionais ou industrializados. Diante do *

Resumo não sigiloso da Atividade em Inglês:

The use of plants for medicinal purposes is a tradition in many cultures around the world, from ancient times. Even today, the popular knowledge provides information about medicinal properties of many species, side and adverse effects, and instructions for its cultivation and preparation for use in treating diseases. Although knowledge of traditional medicine is not as intensely widespread anymore, the tradition of using medicinal plants persist in the population, which often makes use of herbal

Palavra(s)-chave:

Extrato nebulizado, estudo de compatibilidade, Ximenia americana L., comprimido, controle da qualidade,

Palavra(s)-chave em inglês:

Nebulized extract, compatibility study, Ximenia americana L., tablet, quality control, chemical marker, phyt

Período das Atividades:

Data Início: 03/2014

Data término: 02/2016 *

☐ **Ainda não iniciado ou em execução**

Equipe

Adicionar + *

Nome Completo	Documento	Instituição	Nacionalidade	
Ana Cláudia Dantas de Medeiros	92956467468	Universidade Estadual da Paraíba	Brasil	

Cleildo Pereira Santana	06753991492	Universidade Estadual da paraíba	Brasil	

Sobre o Componente do Patrimônio Genético acessado

Adicionar + *

Tipo de Componente	Espécie	
Fauna	Ximenia americana	

Parceria com instituição Nacional

Adicionar +

Parceria com instituição sediada no exterior

Adicionar +

Envio de Amostra que Contenha Patrimônio Genético ao Exterior

Adicionar +

Resultados Obtidos

Adicionar +

Retificação de Cadastro

Adicionar +

Solicitar Atestado de Regularidade

Adicionar +

* **Campos Obrigatórios.**

Termos de uso do SisGen:

Ao realizar este cadastro no SisGen, o usuário reconhece e declara:

I. Ter conhecimento da legislação pertinente, em especial da Lei nº 13.123/2015, e de seus regulamentos;

II. estar ciente do compromisso de não revelar informação reconhecida como sigilosa a que vier ter conhecimento pelo SisGen, sob pena de responsabilização civil, penal e administrativa, conforme disposto na legislação vigente (Arts. 153, 154 e 325 do Código Penal Brasileiro - Decreto-Lei n° 2.848/1940); e

III. ter conferido e ser o detentor dos dados acima informados, respondendo pela sua veracidade, e que os documentos anexados são fac símile dos originais e estarão disponíveis para conferência pelos órgãos competentes, sempre que solicitado, sob pena prevista nos Arts. 299, 307 e 308 do Código Penal Brasileiro(Decreto-Lei n°2.848/1940).

☑ Eu li e concordo com os termos de uso.

(http://www.acessoainformacao.gov.br/) (http://www.brasil.gov.br/)

Desenvolvido por Glück Informática (http://www.gluckinformatica.com.br/)

Voltar para o topo
